# Atypical Pattern of the Intrahepatic Biliary Duct on Magnetic Resonance Cholangiopancreatography in a Tertiary Care Centre: A Descriptive Cross-sectional Study

**DOI:** 10.31729/jnma.7658

**Published:** 2022-11-30

**Authors:** Pragya Shrestha, Dil Islam Mansur, Subindra Karki, Sheprala Shrestha, Sunima Maskey, Roshan Chaudary

**Affiliations:** 1 Department of Anatomy, Kathmandu University School of Medical Sciences, Dhulikhel, Kavre, Nepal; 2 Department of Radiodiagnosis, Dhulikhel Hospital, Dhulikhel, Kavre, Nepal

**Keywords:** *bile ducts*, *liver*, *magnetic resonance cholangiopancreatography*

## Abstract

**Introduction:**

The liver possesses different patterns of intrahepatic duct confluences. Even though the typical pattern of the intrahepatic biliary duct is common, atypical variations are also frequently witnessed. The knowledge of the atypical intrahepatic biliary confluences is inevitable during hepato-biliary surgery to reduce post-operative complications. The aim of the study was to find out the prevalence of the atypical pattern of intrahepatic biliary duct pattern on magnetic resonanace cholangiopancreatography in a tertiary care centre.

**Methods:**

This descriptive cross-sectional study was done in a tertiary care centre from 5 December 2021 to 25 June 2022. Ethical approval was obtained from the Institutional Review Committee [Reference number: KUSMS/IRC (248/2021)]. The branching patterns of the atypical intrahepatic biliary pattern were observed in Magnetic resonance cholangiopancreatography images and were classified. Convenience sampling was used. Point estimate and 95% Confidence Interval were calculated.

**Results:**

Among 199 magnetic resonance cholangiopancreatography images, the atypical intrahepatic biliary duct was observed in 48 (24.12%) (18.18-30.06, 95% Confidence Interval) of the images.

**Conclusions:**

The prevalence of the atypical intrahepatic biliary duct pattern is lower as compared to other studies done in similar settings.

## INTRODUCTION

The liver presents successive branching of the intrahepatic biliary duct (IHD).^1^ In a typical IHD pattern, the right hepatic duct (RHD) is formed by the confluence of the right posterior hepatic duct (RPHD) and the right anterior hepatic duct (RAHD). Concomitantly, RHD and the left hepatic duct (LHD) joined to form a common hepatic duct (CHD).^2^ However, studies show marked atypical IHD patterns. The formation of CHD by the triple confluence of RAHD, RPHD and LHD is common. Besides aberrant drainage of RPHD and accessory ducts are also frequently observed.^3^

The preoperative assessment of IHD variations is essential to formulate a treatment plan and minimise post-operative complications.^4^ The knowledge of atypical IHD patterns is mandatory during liver transplantation, liver resections and cholecystectomy.^5^

The aim of the study was to find out the prevalence of atypical patterns of the intrahepatic biliary duct on magnetic resonance cholangiopancreatography in a tertiary care centre.

## METHODS

A descriptive cross-sectional was performed in the Kathmandu University School of Medical Sciences from 5 December 2021 to 25 June 2022. The data was collected after ethical approval from the Institutional Review Committee [Reference number: KUSMS/IRC (248/2021)]. Data was collected from magnetic resonance cholangiopancreatography (MRCP) images of patients indicated for various clinical investigations in the Department of Radio-diagnosis Dhulikhel Hospital. MRCP with IHD pattern with at least second order branching could be clearly seen were included in the study. Images with pathological conditions were excluded from the study. Convenience sampling was used. The sample size was calculated by using the following formula:


$$\begin{array}{ll} \text{n} & ={\text{Z}}^{2}\times \frac{\text{p}\times \text{q}}{{\text{e}}^{\text{2}}}\\ & =1.96^{2}\times \frac{0.5\times 0.5}{0.07^{2}}\\ & =196 \end{array}$$

Where,

n= minimum required sample size
Z= 1.96 at 95% Confidence Interval (CI)
p= prevalence taken as 50% for maximum sample size calculation
q= 1-p
e= margin of error, 7%

The calculated sample size was 196. However, the study was done in 199 images. Data were obtained from a 1.5 T Philips Magnetic Resonance Imaging (MRI) scanner. The routine department protocol was followed for the MRCP examinations. Patients were thoroughly screened as per the department guidelines. Confidentiality of the data was maintained.

The radiologist visually analysed the images to determine the IHD variations. The branching patterns of IHDs were classified as one of seven types. Type 1 was considered a typical pattern whereas others were categorised as atypical patterns.^5^

The atypical variations were tabulated and photographed for the record. Data were entered and analysed in IBM SPSS 16.0. Point estimate and 95% CI were calculated.

## RESULTS

The study was conducted on 199 MRCP images out of which the atypical intrahepatic biliary duct was observed in 48 (24.12%) (18.18-30.06, 95% CI). Among atypical patterns, type 2 was detected in 29 (60.42%) followed by type 3A in 11 (22.92%). Other aberrant drainages of RPHD, type 3B, and 3C were seen in 3 (6.25%)and 1 (2.08%) images respectively. Moreover, in 1 (2.08%) of the images type 4 and type 5A were seen.

Finally, 2 (4.17%) of the cases presented unclassified variations (Table 1).

**Table 1:** Frequency of anatomical variations of biliary ducts (n= 48).

Anatomical variations	n (%)
Type 2: Triple confluence, RAHD, RPHD, and LHD join to form CHD	29 (60.42)
Type 3A: Drainage of the RPHD into LHD	11 (22.92)
Type 3B: Drainage of the RPHD into CHD	3 (6.25)
Type 3C: Drainage of RPHD into cystic duct	1 (2.08)
Type 4: Drainage of RHD into cystic duct	1 (2.08)
Type 5A: Accessory duct drainage into CHD	1 (2.08)
Type 7: Unclassified or complex variation	2 (4.17)

**Figure 1:**
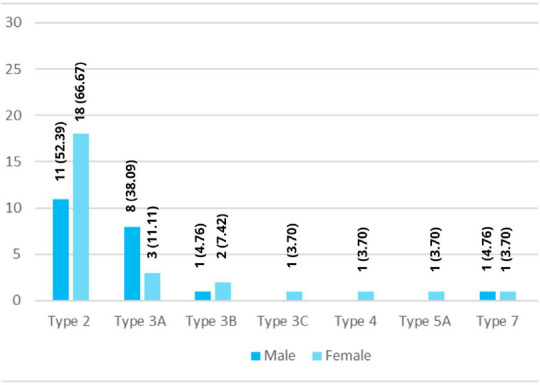
Gender-based anatomic variations of atypical biliary ducts (n= 48).

Out of the total, type 2 anatomic variation was found in 11 (52.39%) male and 18 (66.67%) female (Figure 1).

## DISCUSSION

The academic community has explored the anatomic variations in branching patterns of IHD and determined its frequencies.^5-7^ The present study investigates atypical IHD patterns and their frequencies which is crucial in treatment planning for hepatic surgeries. The typical pattern of IHD confluence is observed in the majority of the studies as seen in the present study.^8^ The atypical pattern of IHD was present in 24.12% which is less than the studies conducted in different parts of Nepal.^8,9^

Among the atypical variations triple confluence (type 2) was the most common pattern which was followed by the type 3A pattern. Likewise, a study among the South Indian population and the Iranian population documented similar findings.^10,11^ The present study contradicts the various studies where type 3A was the most common followed by type 2.^7,12^

A study among the Nepalese population reported triple confluence in the highest percentage which agrees with the present study.^13^ The present study reported type 2 IHD as the most common variation among both males and females which is in congruence with the published study.^13^ In this pattern of triple confluence, RHD is absent and possesses two or more orifices of RAPD, RPHD, and LHD in the plane of transection of the liver graft.^9^ Thus, during liver transplantation, biliary reconstruction of these variants is complicated and technically difficult as it requires multiple anastomoses.^14^

Other clinically relevant anatomic variants in which the RPHD drains into the LHD (type 3A) were present in 22.92% of the images. This atypical variation ranks as the second most common type of variation similar to the study among the Odisha population.^10^ Nonetheless, another study among the Nepalese population revealed this variant only in 3.3% of cases.^9^ In this variant, RPHD may be inadvertently ligated during left hepatectomy which may produce biliary cirrhosis of segments VI and VII.^15^ A cadaveric study in Athens has reported aberrant insertion of RPHD into the CHD (type 3B) in 2.74%.^7^ Comparable to this, the present study also reported this variant in 2.08%. Whereas the previous studies documented this variant up to 6.1%.^4^

This study observed RPHD draining into the cystic duct (type 3C) in 2.08% which supports the published study.^5^ However, a study reported the absence of type 3C variation.^9^ The low insertion of RPHD into CHD (type 3B) and cystic duct (type 3C) variations increase the risk of damage to RPHD during laparoscopic procedures.^6^ This aberrant duct may undergo accidental transection or ligation, subsequently leading to bile leakage or obstruction of segments VI and VII.^17^

Even though a study reported the absence of RHD draining into the cystic duct (type 4), the present study reported this variant in 2.08%.^13^ Other rare variants; accessory hepatic duct with type 5A was present in 2.08% and complex variation of type 7 was present in 4.17%. However, studies have reported more frequencies of accessory ducts.^5,18^ Although an accessory duct may not pose many problems in surgery one has to carefully identify this to prevent postoperative complications. An atypical pattern of the confluence of IHD is frequently appreciated in MRCP images. Among the atypical variants, triple confluence and RPHD draining to LHD are common. Moreover, there are also other IHD variants evident in the study. Precise evaluations of this biliary anatomic variation are inevitable for performing surgery and to avoid serious post-surgical complications.

This study has certain limitations. Firstly, the study was confined to the images of individuals visiting Dhulikhel Hospital and thus could not be a representative study of the whole country. Besides this, the sample size was limited. Further studies with a large sample size including the different geographical regions of Nepal need to be performed.

## CONCLUSIONS

The prevalence of the atypical IHD pattern is less as compared to other studies done in similar settings. Preoperative identification of this variant can modify the treatment plan, thus minimising post-operative complications.
